# Whole-body vibration as an effective sports competition warm-up: a randomized controlled trial in a young and healthy population

**DOI:** 10.3389/fphys.2025.1722108

**Published:** 2026-01-14

**Authors:** Alessandra Amato, Luca Petrigna, Paulo Roberto dos Santos Amorim, Xu Wenxin, Giuseppe Musumeci

**Affiliations:** 1 Department of Biomedical and Biotechnological Sciences, Section of Anatomy, Histology and Movement Science, School of Medicine, University of Catania, Catania, Italy; 2 Department of Physical Education, Federal University of Viçosa, Viçosa, Brazil; 3 School of Physical Education and Sports Science, Fujian Normal University, Fuzhou, China; 4 Research Center on Motor Activities (CRAM), University of Catania, Catania, Italy

**Keywords:** countermovement jump, reaction time, surface electromyography, warm-up, whole-body vibration

## Abstract

**Introduction:**

The long-term effect of training with Whole-Body Vibration (WBV) has been documented to have benefits on health, but its acute role in performance remains unclear. This study evaluated whether a 5-minute WBV warm-up could serve as an effective warm-up to optimize sport performance.

**Methods:**

Ninety-three participants healthy, active but not professional athletes, (23.6 ± 5.7 years) were randomized into three groups: control (CG), sham vibration group (SVG), and vibration group (VG). All participants completed tests at two time points (T0 and T1): countermovement jump (CMJ) the primary outcome, reaction time (RT), reactive strength index (RSI), and sit-and-reach (SRT). During assessments, superficial electromyography (sEMG) was recorded to explore potential neuromuscular changes associated with the interventions. Between T0 and T1, the VG performed a 5-minute WBV warm-up, the SVG completed the same warm-up without vibration, and the CG remained at rest.

**Results:**

VG improved in the primary outcome, CMJ (p < 0.01), SRT (p < 0.01), and RT (p < 0.01), while RSI remained unchanged (p > 0.05). SVG showed similar improvements (CMJ (p < 0.01), SRT (p < 0.01), and RT (p < 0.01), RSI (p > 0.05), whereas CG experienced a decline in RSI (p < 0.05). Between-group contrasts at the post-test endpoint for the primary outcome (CMJ) showed no statistical significance (all p > 0.05), a finding consistent across all other variables.

**Conclusion:**

The proposed 5-minute warm-up protocol, whether performed with or without whole-body vibration, effectively enhanced performance in sport-related tests without inducing a performance decrease. However, WBV did not provide additional benefits over the same warm-up performed without vibration.

## 1 Introduction

Exercise with Whole-Body Vibration (WBV) refers to training on a vibrating platform that delivers varying oscillation frequencies to the full body (Kim, 2018). It is considered to induce neuromuscular system adaptation by activating sensory receptors, such as the muscle spindles in tendons and muscles affected by vibration (Kim, 2018). Increased muscle spindle sensitivity could improve neuromuscular response (Feland et al., 2021). WBV improves recruitment thresholds for motor units and coordination between agonist and antagonist muscles (Allam et al., 2024). Several longitudinal studies have demonstrated the effects of long-term training using this method on postural balance and the risk of falls, as well as osteoporosis prevention and treatment (Chan et al., 2013), and low back pain in neurologic rehabilitation for reducing spasticity (Huang et al., 2017). In addition, WBV was engaged in elite athlete training and injury recovery, as well as for simple fitness purposes, by healthy individuals who reported remarkable improvements in muscle strength (Tan et al., 2023) and body composition after chronic vibration exposure.

However, there are conflicting results on the acute effect of WBV on performance. The lack of a standard operating procedure for WBV training (Petrigna et al., 2024), the lack of clarity in the mechanisms of muscle electrical activity involved, and the different skills assessed do not give information on the possible usefulness of this tool for pre-competition activation.

Feland and colleagues did not detect improvement in jumping performance or in electromechanical delay after 5 min of WBV stimulation, alternating 60 s stimulation with 60 s rest for 10 min (Feland et al., 2021). Instead, Annino and colleagues improved jumping performance and neuromuscular activity by performing the same protocol volume and ratio between exercise and recovery in a similar population but with different set vibration frequencies (Annino et al., 2017). The vibration frequencies were 26 Hz (displacement of 3.6 mm) for the study of Feland and colleagues and 35 Hz (displacement of 5 mm) for Annino and colleagues. In both cases, the participants were stationary on the platform in the half-squat position during the vibration protocol.

Previous studies demonstrate the effectiveness of modified warm-ups in improving performance in open-skill sports, such as jump height, through long-term protocols with relatively extended durations (15–20 min) (Fuchs et al., 2020). However, there is limited evidence from studies showing the acute effect of a short differential warm-up combined with WBV on the performance of these disciplines.

In the last 10 years, previous research has hypothesized using WBV as a warm-up for a sports competition (Chang et al., 2019). However, to date, the results are inconsistent and partial. These studies do not simultaneously analyze the effects on the performance of all the skills necessary for a sports competition, such as power, reaction time, flexibility, and reactive strength of leg muscles. This limits the understanding of the effect of WBV on specific disciplines or single skills. These motor skills are necessary to achieve maximum performance in all “open skills” sports, characterized by a dynamic environment where conditions could change at any time (Heilmann et al., 2022). In addition, the mechanisms of action, such as the muscle’s electrical activity, are not properly clarified. Duc and colleagues observed no differences in electromyography (EMG) activity after preconditioning squat exercise with the WBV in cycling sprint performance (Duc et al., 2020). These results agreed with Barnes and colleagues, where the EMG did not differ from pre to post-warm-up with the WBV (Barnes et al., 2017). The protocols used in these studies vary from 30 s to 5 min with static or dynamic positions during stimulation. However, in all the protocols, WBV stimulation is never continuous but interrupted by recovery without vibration, which could affect the results. Consequently, the present study aimed to combine typical warm-up exercises of an open-skill sport, with continuous vibrating platform stimulation for 5 min. We wanted to study its effects by monitoring the effect on EMG activation of agonist and antagonist muscles. Thus, we propose a standard operating procedure for warm-up with WBV before competition.

While all the assessed skills (reaction time, flexibility, and reactive strength) are relevant to open-skill sports performance, the countermovement jump (CMJ) was selected as the primary outcome for this randomized controlled trial. The CMJ is a well-established, valid, and reliable measure of lower-limb explosive power and neuromuscular readiness (Claudino et al., 2017). In open-skill team sports such as basketball, volleyball, and soccer, vertical jump height is a critical and frequently performed action, directly linked to pivotal moments in competition (e.g., changes of direction, rebounding, blocking, heading). Consequently, an effective warm-up should demonstrably enhance this fundamental athletic quality without inducing fatigue. The secondary outcomes (reaction time, sit-and-reach, and reactive strength index) provide a comprehensive profile of other essential physical attributes affected by the warm-up intervention.

Understanding whether WBV has an established positive or negative effect could help to understand whether WBV can be used as a warm-up to optimize performance by requiring less time and space. Therefore, the objective of this study is to evaluate the effect of a 5-minute continuous WBV warm-up on reaction time, leg muscle power, flexibility, and leg muscle reactive strength. Exploratory sEMG measures were also collected to examine potential neuromuscular associations.

## 2 Materials and methods

### 2.1 Study design

One hundred and twenty participants were invited to take part in the study. They were contacted via email using the institutional mailing list of physical education students at the University of Catania (Catania, Italy). The email described the study design, evaluation procedures, and inclusion/exclusion criteria. Participants were instructed to read the information and reply to confirm their interest and eligibility.

Upon confirmation of eligibility by reply to the mail, participants were randomly assigned to one of the three groups: Vibration Group (VG), Sham Vibration Group (SVG), or Control Group (CG). Randomization was conducted using a computer-generated block randomization procedure to ensure balanced allocation across the three groups. A researcher (AA) generated the randomization sequence in blocks of three, using random permutations of group assignments (e.g., VG–SVG–CG, SVG–CG–VG, etc.). Group assignment was concealed using a centralized list with sequential participant codes by another researcher (LP) who was blinded to the assignment of codes to individual participants and who did not participate in the recruitment process. Participants were partially blinded due to the nature of the study design: the allocation was revealed only after each participant confirmed eligibility and enrollment and went to the assessment day. They were not aware of the study hypothesis, so as not to influence their performance. The blindness of the evaluators was maintained in the assignment of groups and during the evaluation section, because one operator performed the pre-test tests and another operator performed the post-test tests, so that they could not know and therefore could not be influenced by the subject’s pre-test value or by the different pre- and post-test values between the groups. Thus, while participants could not be fully blinded to the intervention due to the perceptible nature of vibration, assessors were blinded to group allocation, and participants were blinded to the study hypothesis to minimize expectancy effects.

On the day of evaluation, a questionnaire was proposed to confirm their eligibility, to collect personal information, and details about their habits, such as physical activity and dominant limb.

A maximum voluntary contraction (MVC) evaluation for tibialis anterior (TA) and gastrocnemius lateralis (GL) was performed to evaluate the % of muscle activation during the performance. All participants performed a sport skill-related battery test evaluation at T0, before the experimental procedure, and at T1, after the experimental procedure. Each group was evaluated on separate days.

**FIGURE 1:**
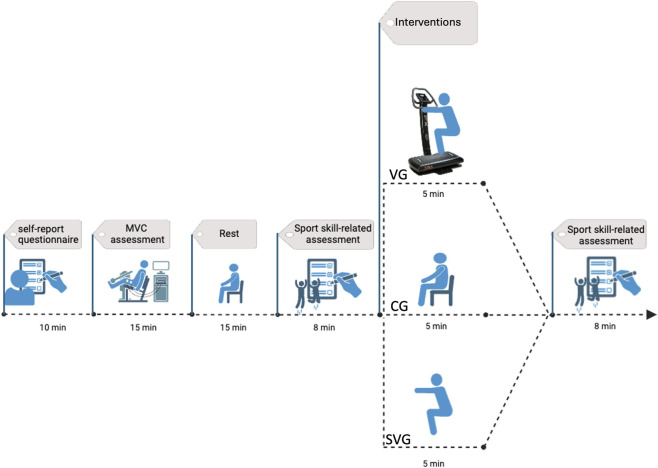
Study design and timeline. The protocol began with the administration of a self-report questionnaire. Following this, participants underwent a Maximum Voluntary Contraction (MVC) assessment, followed by a rest period. A sport skill-related assessment was then conducted. Subsequently, participants were divided into a VG (vibration group), SVG (sham vibration group), or CG (control group) and performed a vibration warm-up, a sham vibration warm-up, or no warm-up (rest), respectively, for 5 min. Finally, the session concluded with a post-test sport skill-related assessment. Key durations for assessments, interventions, and rest periods are indicated. The total approximate duration of the session was 38 min.

The VG performed a 5-minute warm-up on the vibrating platform turned on between T0 and T1 assessments; the SVG performed the same 5-minute warm-up protocol as the VG but without vibration between T0 and T1 assessments. The CG rested between T0 and T1 without performing any warm-up. The data collection lasted about 1 h for each participant. Figure 1 shows the design and timeline of the study. Adverse events and unintended effects were systematically monitored throughout the study. After each testing session, participants were verbally asked by a researcher whether they had experienced any discomfort, pain, dizziness, nausea, or other unusual sensations during or after the warm-up protocol. Responses were recorded using an Excel file with “no” if no adverse events were registered or a qualitative answer if adverse events occurred. In addition, participants were asked to send an email to the researcher within 24 h after their session to inquire about any delayed symptoms.

### 2.2 Participants

Participants were included if they met the following criteria: aged 18–45 years (young adults); engaging in structured physical activity for a minimum of 3 h per week over the past 6 months; Free from any musculoskeletal pain or injury for at least 6 months before the study. Participants were excluded if they had any history of major surgery or fracture in the lower limbs or spine within the preceding 12 months; any known neurological, vestibular, or cardiovascular disorder that could impair balance or be exacerbated by WBV (e.g., epilepsy, severe osteoporosis); presence of any implanted electronic medical device (e.g., pacemaker, defibrillator, cochlear implant); current use of medications known to affect balance or neuromuscular function (e.g., psychotropics, muscle relaxants); Pregnancy. An information questionnaire was submitted to our participants to gather this information. The questionnaire was set up using Google Forms to ensure that both the questions were understandable to participants and the answers were understandable to researchers. The study was conducted following the Declaration of Helsinki and approved by the scientific committee of the “Research Center on Motor Activities” of the University of Catania (Catania, Italy), CRAM-033-2023, 15/03/2023. Participants were informed of the risks and benefits of participation in this study, and participants provided their written informed consent to use their data anonymously before the study. The participants were advised that they could withdraw from the study at any time and for any reason. They did not receive any kind of compensation. All participants who provided informed consent and were randomized constituted the intention-to-treat (ITT) population. The primary efficacy analysis was conducted on a modified intention-to-treat (mITT) population, which included all randomized participants who completed at least the baseline assessment.

### 2.3 Maximum voluntary contraction assessment

MVC measurements were performed with the participant sitting in a chair that allowed an angle between the thigh and the leg of 90° and a neutral ankle position (90°).

**FIGURE 2:**
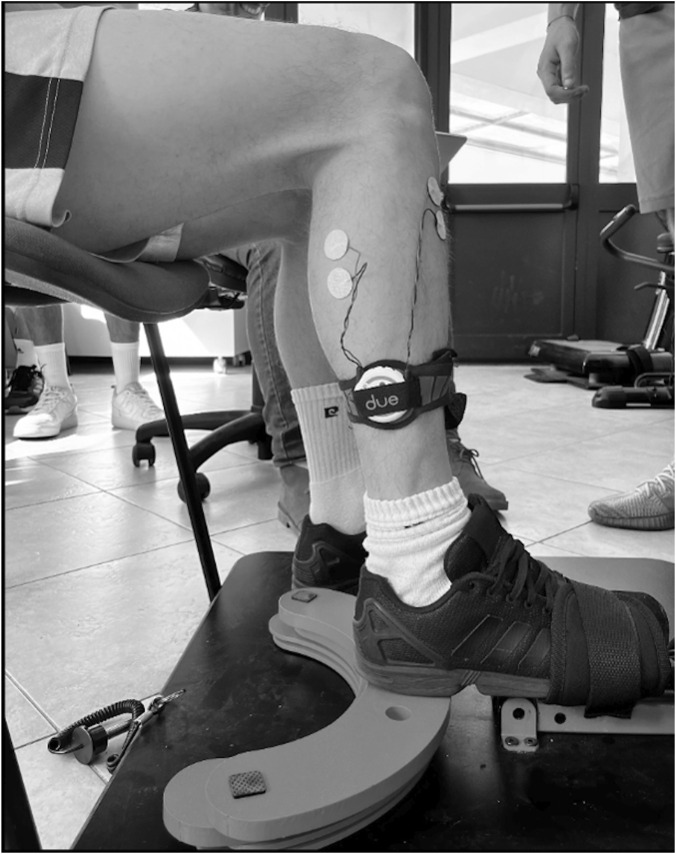
TA MVC evaluation setting. The figure shows a study participant performing the MVC for TA. The image is a photograph taken with the participant’s consent during the preparatory phase of the MVC of the dominant limb (right leg) and shows the exact placement of the electrodes of the EMG device: two in the lateral gastrocnemius and two in the anterior tibialis.

Participants were instructed to perform three dorsiflexion MVCs (Figure 2) to record maximal sEMG activity from the TA and three plantar flexion MVCs to record maximal sEMG activity from the soleus. Participants had to reach the maximal effort gradually in three seconds, keep the effort for 2 seconds, and relax. Each contraction was followed by at least 2 min of rest. The higher score from the three trials for each muscle was used as the MVC reference value for normalization. During the tests, the leg was constrained to the floor using a band tied to the Flyconpower conical machine (Flyconpower SRL, Cuneo, Italy), as shown in Figure 2.

### 2.4 Surface electromyography (sEMG) analysis

One investigator with previous experience with sEMG collected all the data with this instrument. The application of the electrodes followed the SENIAM’s recommendation (Hermens et al., 2000). The electrodes on the TA and GL were applied following previously published guidelines (Rainoldi et al., 2004).

sEMG analysis was performed during the test battery at T0 and T1 following a procedure previously standardized and published by our research group (Petrigna et al., 2025a). EMG signal was recorded using a 2-channel EMG (Dualite, OT Bioelettronica, Turin, Italy), the recording bandwidth of 10–500 Hz with a sampling frequency of 2,048 Hz. Bipolar electrodes 24 mm with concentric connector (5pcs), Ag/AgCl surface (Spes Medica, Genova, Italy) were adopted. A 0.5-cm interelectrode distance was adopted. Before the application of the electrodes, the skin was shaved, washed with alcohol, and abraded to prevent the cables from swinging and from causing movement artifacts. The data were visualized and analyzed through a personal computer using OT BioLab+ software (version 1.5.9, OT Bioelettronica, Torino, Italy). EMG signal of the jump was first root mean square (RMS) converted. The average RMS was integrated and expressed as a function of time expressed in millivolts (mVs). The EMG analyses from the TA and GL muscles during the performance were averaged and converted into a percentage of MVC. The percentage of MVC activation for TA (TA MVC% %) and GL (GL MVC% %) was used to calculate the agonist/antagonist muscle ratio (GL/TA ratio). In the countermovement jump (CMJ), the gastrocnemius lateralis (GL) acts as a primary agonist during the plantar flexion phase, contributing substantially to vertical force production and propulsion. The tibialis anterior (TA) serves as an antagonist during propulsion but plays a critical role in dorsiflexion and ankle stabilization during the countermovement and landing phases. The balance between agonist and antagonist activation, reflected in the GL/TA ratio, provides insight into neuromuscular coordination and movement efficiency during explosive lower-limb tasks (Van Hooren and Zolotarjova, 2017).

### 2.5 Sport skill-related battery test evaluation

We simulated in a laboratory setting a Sports skill-related performance by assessing: lower limb power with the countermovement jump test (CMJ) (Glatthorn et al., 2011), selected as the primary outcome; reaction ability by recording lower limb reaction times (RT) (Glatthorn et al., 2011) to a visual stimulus;, reactive strength of leg muscles assessed using the reactive strength index (RSI) (Lehnert et al., 2018), and back muscle chain flexibility with the sit and reach test (SRT) (Ayala et al., 2012).

#### 2.5.1 Countermovement jump test

The CMJ was designated the primary outcome due to its high ecological validity for open-skill sports (Sheppard and Young, 2006). To perform the CMJ, we used the Optojump photocell system (MICROGATE, Bolzano, Italy). This is a validated tool for Estimating Vertical Jump Height (Glatthorn et al., 2011), consisting of one receiver bar and one transmitter bar placed parallel. Before performing the jump, the participant stood in an upright position between the two bars, with the distance between the two feet approximately equal to the width of his shoulders. After the operator’s signal, when he felt ready, from a standing position, the participant performed a maximal vertical jump. They were allowed to perform a free countermovement. They could use his arms freely. Bars were connected to a personal computer where, through OPTOJUMP NEXT PC software (version 1.13.24), jump height was indirectly estimated through the flight time of vertical jumps with an accuracy of 1/1,000 s (1 kHz). After being explained and described, only one jump was performed by the participants so as not to cause fatigue. Height in centimeters was used for the analysis. Before the recorded trial at T0 and T1, participants performed one practice jump to familiarize themselves with the procedure and equipment. The recorded jump was performed after 1 minute.

#### 2.5.2 Sit and reach test

SRT is a reproducible and valid test (Ayala et al., 2012) to evaluate the mobility of the human back muscle chain. The test is performed using a box, which is a standardized instrument. Participants were seated with legs straight on the floor, and the soles of the shoes were in contact with the box. The tool used was the “Baseline 12–1,085” (Fabrication Enterprises, New York, United States of America). From this starting position, after inspiration and during expiration, the participants had to perform a forward trunk flexion with outstretched arms and fingers and reach as far as possible on the number scale above the box in centimeters.

The test was repeated three times, both at T0 and T1, and the average value among the three scores was used for the analysis. Participants performed one practice repetition before the three recorded trials at both T0 and T1 to ensure understanding of the technique and minimize learning effects during data collection.

#### 2.5.3 Reaction time test

RT was recorded with the same tool as CMJ, the Optojump photocell system widely validated tool for assessing reaction time (Mroczek et al., 2011; Bosquet et al., 2009). Furthermore, we had previously tested the specific test setting (Amato et al., 2022) Participants stood between the bars in a half-squat position, looking toward the pc monitor placed in front of them, on which a large red dot was. Whenever the signal turned green, participants had to get both feet off the ground as quickly as possible. RT protocol is a standardized protocol found in the OPTOJUMP NEXT software (version 1.13.24).

The Optojump photocell system recorded the time between the time when the dot turned green and the time when the optical signal between the emitting and receiving bars, which was interrupted by the feet in the starting position, was restored, calculating the reaction time. The dot turned green three times; the best result of the three trials, the lowest time in seconds, was used for the analysis both in T0 and T1. Before the three recorded reaction time trials, participants completed one practice trial to become familiar with the visual stimulus and the required movement.

#### 2.5.4 Reactive strength index

RSI refers to the athletes’ skill to transition from eccentric to concentric muscle contraction during the lengthening-shortening cycle (Lehnert et al., 2018). It demonstrated that RSI has a strong correlation with change of direction speed, a key skill in open-skill sports competition (Young et al., 2015).

RSI was assessed by performing a drop-jump, landing between the two bars of the Optojump photocell system. Participants were instructed to stand with feet shoulder-width apart on a 31 cm box (Myer et al., 2010; Sasaki et al., 2019) and drop down to immediately attempt three maximum jumps for the shortest take-off period consistent with prior studies’ setup (Lehnert et al., 2018).

The RSI was calculated as the ratio between jump height and contact time on the floor recorded by the Optojump photocell system. The average of the three RSI values was considered for the data analysis. Prior to the three recorded drop jumps, participants executed one practice drop jump to familiarize themselves with the landing and immediate rebounding technique.

### 2.6 Whole-body vibration intervention

The VG performed a 5-minute warm-up on the DKN XG5 Vibration Platform (DKN-Technology–Langerode 17/B01, 3460 Bekkevoort), delivering vertical sinusoidal vibration stimulus.

**FIGURE 3:**
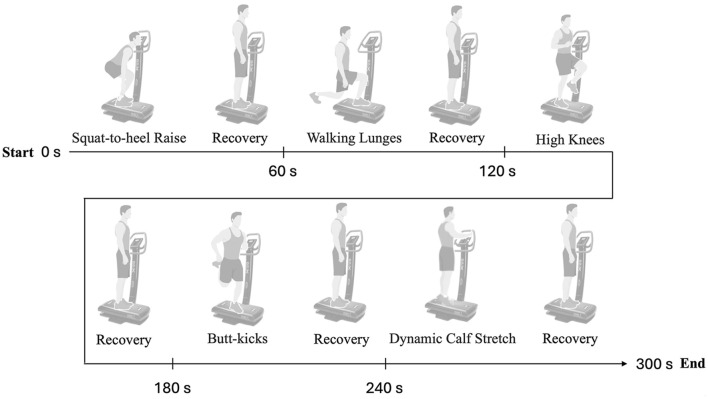
Schematic timeline of the dynamic warm-up protocol. The warm-up consisted of six exercises performed over a total duration of 300 s (5 min). The protocol began at 0 s with Squat-to-heel Raise, followed by a recovery period (120°–130° position between leg and thigh). Subsequent exercises included Walking Lunges and High Knees, each interspersed with designated recovery intervals. The second half of the warm-up comprised Butt-kicks and Dynamic Calf Stretch, also separated by recovery periods. Timepoints for exercise initiation and transition are indicated at 0 s, 60 s, 120 s, 180 s, 240 s, and 300 s (End).

The platform is located inside the laboratory “Research Center on Motor Activities (CRAM)” of the University of Catania, in a room whose temperature is controlled by an air conditioner, and is far from possible light or sound interference. Before starting the warm-up protocol, an assistant demonstrated all the exercises and the recovery phase position. The assistant also demonstrated the rhythm, emphasizing that the intensity of execution should be maximum, i.e., the participant should perform as many repetitions as possible in 30 s for each exercise. A practical familiarization session was not carried out to avoid inducing fatigue, as the assessments were carried out on a single day. An assistant was positioned next to the platform to be ready at any moment to press the stop button and interrupt the vibration. At the beginning of the warm-up, the VG participants stood on the vibration platform and had to look forward and keep their hands on the handrails to prevent slipping on the platform. The protocol consisted of 5 exercises performed for 30 s, alternated with a 30-second recovery during which the participant maintained a thigh bend position of between 120° and 130° for a total of 300 s of warm-up. This standardized warm-up procedure was adapted from Cabarkapa et al. (Cabarkapa et al., 2023). Both during the exercises and recovery, the vibration frequency was 45 Hz displacement of 1.2 mm. The frequency of 45 Hz was selected based on previous evidence suggesting that frequencies above 30 Hz are more effective in inducing acute neuromuscular responses, such as increased muscle spindle activation and facilitation of the tonic vibratory reflex (TVR) (Torvinen et al., 2002; Petrigna et al., 2024). Specifically, studies have demonstrated that frequencies between 35 Hz and 50 Hz enhance vertical jump performance and agonist-antagonist coordination without inducing early fatigue (Annino et al., 2017; Kim, 2018). For instance, Annino et al. (2017) reported significant improvements in jump performance at 35 Hz, whereas protocols using lower frequencies (e.g., 26 Hz) showed no significant effects on these outcomes (Feland et al., 2021). However, the Annino’s protocol lasted 10 min, we opted for 45 Hz with a displacement of 1.2 mm to maximize acute neuromuscular stimulation while maintaining a safe profile suitable for a pre-competition warm-up and for less time: 5 min. Therefore, the SVG performed the same exercises but without vibration: participants belonging to the SVG group had to stand on the switched-off platform and perform 30 s of exercise and 30 s of recovery phase, maintaining a thigh bend position of between 120° and 130° and grasping the handles of the vibrating platform in front of them. The order of the exercises was the same as for the VG and is shown in Figure 3. The warm-up exercise and timing are detailed in Figure 3. After completing all the assessments at T0, the CG participant had to remain seated for 5 min on a chair that maintained an angle of approximately 90° between the leg and thigh and had a backrest to support the back (a situation that simulated that of players on the bench during a match). One minute of recovery time was given after the warm-up, during which participants sat down to allow the operator to position the EMG electrodes.

### 2.7 Statistical analysis

The SPSS ® (IBM ®, Chicago, IL, USA) version 29.0.2.0 software program was used for statistical analysis. The data were expressed as the mean (M) and standard deviation (SD). The significance level was defined as p ≤ 0.05. First, the Shapiro-Wilk test was used to verify the normality of the data. Mixed-design ANOVA (3 conditions x 2 time intervals) was used to analyze the differences within and between groups for each variable, with partial eta squared (η^2^p) reported as the measure of effect size for significant effects. The Bonferroni *post hoc* analyses were used to locate the differences between conditions. We reported the magnitude of between-group differences for all outcome measures using Cohen’s d and its 95% confidence intervals.

An *a priori* sample size calculation was performed using G*Power software (Version 3.1, University of Düsseldorf, Germany). The calculation was based on the results of a previous study (Kim, 2018).

From this data, an effect size f of 0.5 was calculated. To ensure adequate power to detect a more conservative and potentially more realistic effect, we used an effect size f of = 0.3 for our calculation. With an alpha (α) level set at 0.05, a desired power (1-β) of 0.95, a correlation between repeated measurements set at 0.80, and Nonsphericity ε set at 1 for ANOVA within-between group interaction, the analysis indicated that a minimum sample size of 18 participants per group was required.

One-way ANOVA was used to investigate baseline participants’ characteristics between groups.

## 3 Results

**FIGURE 4:**
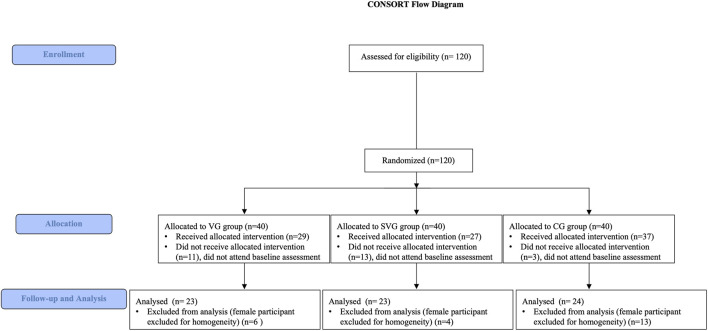
CONSORT flowchart of participant progression through the phases of the randomized controlled trial. The diagram illustrates the enrollment, allocation, follow-up, and analysis of study participants. It details the number of individuals assessed for eligibility, those excluded (with reasons), and those randomly allocated to intervention groups. The flow chart further tracks participants through each study phase (e.g., intervention, follow-up), accounting for any losses or exclusions post-allocation. Finally, it specifies the number of participants included in the primary analysis for each group.

A total of 120 individuals were initially contacted and randomly (ITT population) allocated to one of the three study groups. However, 27 participants (CG: n = 3; SVG: n = 13; VG: n = 11) did not attend the scheduled baseline assessment session and were therefore excluded from the mITT analysis, resulting in a final mITT sample of 93 participants. These dropouts occurred before any data collection and were not due to adverse events or protocol violations. No adverse events or side effects requiring medical attention were anticipated, given the brief, low-intensity nature of the warm-up protocol. Due to the significant imbalance in the number of men and women after the recruitment process, allocation, and follow-up, with female participants not being uniformly distributed across the intervention groups, we were unable to make a comparison between genders to make the sample more homogeneous, and because speed–strength abilities are strongly sex-dependent, the minority group (female n = 23) was excluded from the analysis. The CONSORT flow diagram in Figure 4 shows the participant recruitment and exclusion process.

The final sample consisted of 70 male participants (height: 176 ± 5.9 cm; weight: 75.9 ± 10.3 kg; body mass index: 24.4 ± 3.5; age: 23.6 ± 5.7 years) (Table 1).

**TABLE 1:** Descriptive statistics (mean ± standard deviation) for all measured variables across the three groups.

Variables	VG	SVG	CG	F	p- value
Age (year)	24 ± 7.5	22.5 ± 4.1	24.4 ± 5.2	1	0.366
Height (cm)	175.9 ± 6.5	175.3 ± 5.3	178.1 ± 5.7	1.7	0.197
Weight (kg)	71.5 ± 7	75.6 ± 7.5	80.3 ± 13.3	4.5	0.016
BMI (kg/m^2^)	23.2 ± 2.4	24.7 ± 3	25.4 ± 4.3	3.2	0.052
Weekly exercise (min)	368.2 ± 147.6	365 ± 150	304.5 ± 118.9	1.4	0.266

VG, Vibration group; SVG, Sham vibration group; CG, Control group; BMI, body mass index; min, minutes. “F” refers to the test statistic from the ANOVA used to assess group differences, and “p” indicates the associated probability value. A p-value less than 0.05 is considered statistically significant.

No differences in age, BMI, Height, or minutes per week of exercise were found between the groups. The self-reported questionnaire administered initially showed that n = 23 participants performed sports activity during the week, n = 12 aerobic activity, and n = 35 muscle strength or power exercise. One-way ANOVA showed a significant difference between groups in weight between the VG and control groups (p = 0.016). Table 1 details the one-way ANOVA analysis for the participants’ characteristics.

### 3.1 Sport skill-related battery test evaluation

Results for the primary outcome, the Countermovement Jump (CMJ), are presented first, followed by secondary outcomes.

#### 3.1.1 Countermovement jump test

A main effect of time was found, F (1, 67) = 13.56, p < 0.001, partial η^2^ = 0.17. Participants showed higher elevation scores at T1 (M = 37.60, SD = 9.66) than at T0 (M = 36.00, SD = 9.52). There was no significant time × group interaction, F (2, 67) = 0.20, p = 0.821, partial η^2^ = 0.01, and no between-group effect, F (2, 67) = 0.02, p = 0.981, partial η^2^ = 0.01. This indicates that the improvement in jump height over time was not statistically different between the three intervention groups. Pairwise comparisons showed significant within-group improvements of 1.68 cm (95% CI [0.15, 3.21], p = 0.032, d = 0.45) for VG and of 1.92 cm (95% CI [0.39, 3.45], p = 0.015, d = 0.51) for the SVG (Δ = 1.83, p = 0.006) but not significant in the CG (+ 1.25 cm, 95% CI [-0.24, 2.75], p = 0.099, d = 0.34) (Table 2).

**TABLE 2:** Sport skill-related battery test score for T0 and T1.

Variables	T0 (M ±SD)	T1 (M± SD)	p value
Group VG			
RSI (m/s)	0.7 ± 0.29	0.7 ± 0.30	0.202
RT (s)	0.5 ± 0.09	0.5 ± 0.9	0.019*
CMJ (cm)	39.6 ± 6.46	41.1 ± 6.6	0.032*
SRT (cm)	26 ± 9.2	27.8 ± 9.3	<0.004**
Group SVG			
RSI (m/s)	0.7 ± 0.32	0.6 ± 0.34	0.088
RT (s)	0.5 ± 0.8	0.5 ± 0.7	<0.001**
CMJ (cm)	39.2 ± 6.87	41.1 ± 7.99	0.015*
SRT (cm)	23.1 ± 7.3	26.0 ± 7.05	<0.001**
Group CG			
RSI (m/s)	0.7 ± 0.39	0.6 ± 0.33	0.060
RT (s)	0.5 ± 0.07	0.5 ± 0.8	0.146
CMJ (cm)	39.3 ± 10.06	40.6 ± 8.43	0.099
SRT (cm)	28.8 ± 5.78	30.56 ± 5.26	<0.002*

Descriptive statistic in time and between group of resistance strength index (RSI), reaction time test (RT), countermovement jump test (CMJ) and sit and reach (SRT) test performances recorded in VG (vibration group), SVG (sham vibration group) and CG (control group) before and after (T0 and T1) the respective training protocol performed. P-value refers to within-group ANOVA pairwise comparisons; *p < 0.05; **p < 0.01.

#### 3.1.2 Sit and reach test

There was a significant main effect of time, F (1, 67) = 42.11, p < 0.001, partial η^2^ = 0.386. Performance improved at T1 (M = 28.11, SD = 0.88) compared to T0 (M = 25.95, SD = 0.90). The interaction between time and group was not significant, F (2, 67) = 1.36, p = 0.263, partial η^2^ = 0.039, nor were the between-group effects, F (2, 67) = 2.89, p = 0.063, η^2^ = 0.079. Within-group Pairwise comparisons revealed a significant improvement from T0 to T1 in the VG (Mean Diff. = −1.75, p = 0.004, d = 0.19, 95% CI = [0.59, 2.91]), in the SVG (Mean Diff. = −2.94, p < 0.001, d = 0.42, 95% CI = [1.78, 4.10]), and in the CG (Mean Diff. = −1.79, p = 0.002, d = 0.33, 95% CI = [0.65, 2.92]) (Table 2).

#### 3.1.3 Reaction time test

A repeated-measures ANOVA showed a significant main effect of time on RT, F (1, 67) = 18.03, p < 0.001, partial η^2^ = 0.21. Participants responded faster at T1 (M = 0.493, SD = 0.087) compared to T0 (M = 0.520, SD = 0.091).

The time × group interaction was not statistically significant, F (2, 67) = 1.03, p = 0.362, partial η^2^ = 0.03, suggesting similar trends across groups. Between-subject effects of group were not significant, F (2, 67) = 0.04, p = 0.965, partial η^2^ = 0.001. Pairwise comparisons showed a significant within-group reduction in RT from T0 to T1 for SVG (95% CI [-0.065, −0.017], p < 0.001, d = −0.55) and VG (95% CI [-0.053, −0.005], p = 0.019, d = −0.33). No significant change was observed in the Control group (95% CI [−0.041, 0.006], p = 0.146, d = −0.22) (Table 2). No significant between-group differences were found at either time point (all p > 0.05).

#### 3.1.4 Reactive strength index (RSI)

The main effect of time was significant, F (1, 67) = 8.05, p = 0.006, partial η^2^ = 0.107, with RSI decreasing from T0 (M = 0.696, SD = 0.041) to T1 (M = 0.63, SD = 0.039). The time × group interaction was not significant, F (2, 67) = 0.099, p = 0.906, nor were group effects, F (2, 67) = 0.106, p = 0.899, partial η^2^ = 0.003.

Given the non-significant interaction, the primary evidence for an intervention effect is derived from the between-group contrasts at the post-test endpoint, adjusted for multiple comparisons using the Bonferroni method. None of these comparisons reached statistical significance; between-group effects with effect sizes and 95% CIs are shown in Table 3.

**TABLE 3:** Between-group post-test contrasts for all outcome measures.

Variables	Comparison (group I vs. Group J)	Mean difference (I-J)	95% CI for difference	p-value	Cohen’s d [95% CI]	95% CI for Cohen’s d
SRT (cm)	CG vs. SVG	4.5	[−0.74, 9.82]	0.116	0.6	[−0.16, 1.36]
	CG vs. WB	2.8	[−2.49, 8.08]	0.594	0.4	[−0.39, 1.13]
	SVG vs. WB	−1.7	[−7.08, 3.60]	1.000	−0.2	[−0.99, 0.53]
CMJ (cm)	CG vs. SVG	−0.5	[−6.08, 5.00]	1.000	−0.1	[−0.73, 0.60]
	CG vs. VG	−0.7	[−6.19, 4.89]	1.000	−0.1	[−0.75, 0.58]
	SVG vs. VG	−0.1	[−5.71, 5.48]	1.000	−0.02	[−0.68, 0.65]
RT (s)	CG vs. SVG	0.01	[−0.050, 0.067]	1.000	0.1	[−0.56, 0.77]
	CG vs. VG	0.000	[−0.059, 0.058]	1.000	0.001	[−0.66, 0.66]
	SVG vs. VG	−0.01	[−0.068, 0.050]	1.000	−0.1	[−0.78, 0.55]
RSI	CG vs. SVG	0.02	[−0.210, 0.255]	1.000	0.1	[−0.60, 0.73]
	CG vs. VG	−0.03	[−0.262, 0.203]	1.000	−0.1	[−0.76, 0.57]
	SVG vs. VG	−0.05	[−0.287, 0.183]	1.000	−0.2	[−0.82, 0.51]

RSI, Resistance strength index; RT, reaction time test; CMJ, countermovement jump test; SRT, sit and reach; VG, vibration group; SVG, sham vibration group; and CG, control group.

### 3.2 Electromyographic (EMG) activity during the sport skill test battery

The main effect of time for the TA activation was not statistically significant, F (1, 56) = 0.618, p = 0.435, partial η^2^ = 0.011, indicating that TA did not change significantly from T0 to T1 when collapsing across groups. The time × group interaction was not statistically significant, F (2, 56) = 1.760, p = 0.181, partial η^2^ = 0.059. This suggests that the change in TA activation over time was not different across the three groups. The main effect of group was not statistically significant, F (2, 56) = 1.559, p = 0.219, partial η^2^ = 0.053, indicating no overall difference in TA activation between the groups when data from both time points were combined.

The main effect of time for the GL activation was not statistically significant, *F* (1, 54) = 1.954, p = 0.168, partial η^2^ = 0.035, indicating that the overall GL contribution did not change significantly from T0 to T1. The time × group interaction was non-significant, *F* (2, 54) = 2.775, p = 0.071, partial η^2^ = 0.093; The main effect of group was not statistically significant, *F* (2, 54) = 0.431, p = 0.652, partial η^2^ = 0.016, indicating no overall difference in GL contribution levels between the groups when data from both time points were combined.

To explore the near-significant interaction, analyses of simple effects were conducted. The simple effect of time was significant only for the VG, *F* (1, 54) = 5.426, p = 0.024, partial η^2^ = 0.091. Post-hoc tests revealed a significant decrease in GL contribution from T0 (Mean = 58.16, SD = 33.85) to T1 (Mean = 48.84, SD = 26.08), p = 0.024. No significant within-group changes over time were found for the control (p = 0.328) or SVG (p 0.261) groups.

No significant between-group differences were found at T0 or T1 in the pairwise comparisons (all p > 0.05) (Table 4).

**TABLE 4:** EMG analysis percentage of muscle activation during the Sport Skill Test Battery.

Variables	T0 (M ±SD)	T1 (M± SD)	p value
Group VG			
TA MVC (%)	30.4 ± 10	29.8 ± 5.79	0.931
GL MVC (%)	58.2 ± 33.85	48.8 ± 26.09	0.024*
GL/TA ratio	2.2 ± 1.44	1.7 ± 0.82	0.032*
Group SVG			
TA MVC (%)	31.0 ± 9.40	43.1 ± 7.09	0.049
GL MVC (%)	48.9 ± 18.03	44.5 ± 21.16	0.261
GL/TA ratio	1.8 ± 1.15	1.5 ± 0.91	0.103
Group CG			
TA MVC (%)	41.5 ± 17.94	38.4 ± 10.35	0.630
GL MVC (%)	47.6 ± 20.48	51.7 ± 24.62	0.328
GL/TA ratio	1.2 ± 0.46	1.34 ± 0.77	0.364

Mean and standard deviation of percentage of MVC activation for Tibialis Anterior (TA MVC %), Gastrocnemius Lateralis (GL MVC %) and agonist/antagonist muscle ratio (GL/TA ratio) recorded in VG (vibration group), SVG (sham vibration group) and CG (control group) before and after (T0 and T1) the respective training protocol performed. P-value refers to within-group ANOVA pairwise comparisons; *p < 0.05.

The main effect of time was not statistically significant for the GL/TA ratio, *F* (1, 53) = 2.819, p = 0.099, partial η^2^ = 0.051, indicating that the GL/TA ratio did not change significantly from T0 to T1 when collapsing across groups; The time × group interaction was not statistically significant, *F* (2, 53) = 2.736, p = 0.074, partial η^2^ = 0.094, suggesting that the change in the GL/TA ratio over time was not significantly different across the three groups; The main effect of group was non-significant, *F* (2, 53) = 2.572, p = 0.086, partial η^2^ = 0.088. Despite the non-significant interaction, planned pairwise comparisons were examined to investigate simple effects. Analyses of the simple effects of time within each group revealed a significant decrease in the GL/TA ratio for the VG from T0 (Mean = 2.16) to T1 (Mean = 1.70), p 0.032. No significant within-group changes over time were found for the control (p = 0.364) or SVG (p = 0.103) groups.

## 4 Discussion

This study investigates the feasibility of employing the WBV as a warm-up before sports competition, with a primary focus on its effect on explosive lower-limb power as measured by the CMJ. It analyzes its effects on four sports-related skills and includes exploratory sEMG recordings of TA and GL to examine potential neuromuscular patterns associated with the intervention. In general, our results show significant improvement or steady performance in all tests for the VG and the SVG without ever showing significant deterioration, unlike the CG. With no significant between-group differences observed. This indicates that the dynamic warm-up exercises themselves were effective, and the addition of WBV did not yield further significant enhancements. Regarding the primary outcome, both the VG and the SVG showed significant within-group improvements in CMJ height. The absence of a significant time × group interaction suggests that the warm-up protocol itself, rather than the vibration component, was responsible for the observed gains in lower-limb explosive power. The significant improvement observed in both the VG and SVG groups aligns with well-established physiological principles of an effective warm-up. The 5-minute dynamic protocol, comprising exercises such as squat-to-heel raises, lunges, and high knees, was designed to elevate muscle temperature and core body temperature. An increase in intramuscular temperature enhances the efficiency of metabolic reactions, reduces the viscous resistance of tissues, and, crucially, accelerates the rate of force development (RFD) by improving the kinetics of cross-bridge cycling and calcium ion handling (Bishop, 2003; McGowan et al., 2015). As the CMJ is a maximal explosive task highly dependent on RFD, this temperature-mediated effect provides a primary rationale for the performance enhancement seen in both active warm-up groups, independent of vibration.

In addition, WBV above 30 Hz have been shown to activate the response known as tonic vibratory reflex (TVR), which can increase recruitment of the number of motor units, resulting in improved contractile characteristics and muscle strength (Torvinen et al., 2002). This would appear to be due to higher activation of muscle spindles and polysynaptic pathways (De Gail et al., 1966). WBV is known to induce neurogenic adaptation and stimulation in lower limb muscles (Bosco et al., 1999). This could account for the significant increase in CMJ test height in the VG (Table 2). However, Torvinen et al. suggest that this adaptation may be transient. The author records increased jumping performance immediately after WBV, but this is not sustained 60 min after stimulation. The study required participants to do nothing between the first and second measurements (Torvinen et al., 2002). In contrast, if the WBV warm-up protocol is proposed before competition, it could be hypothesized that adaptation and, thus, performance could be maintained by constant muscle activation during the sport competition itself. Another possible explanation linking WBV with the increase in CMJ jump height is its biomechanics. Indeed, the stretch-shortening cycle activates the spinal reflexes during CMJ to achieve the best jump height, unlike the concentric squat jump, which does not have this cycle (Van Hooren and Zolotarjova, 2017). Therefore, it is reasonable to hypothesize that any neurogenic stimulation, like WBV, that increases stretch reflex mechanism sensitivity can influence the CMJ test performance. In our results, the improvement in strength and power observable in CMJ jump height increased in VG, agreeing with previous studies showing improvement on CMJ by administering different protocols of WBV (Cochrane et al., 2010; Annino et al., 2017; Feland et al., 2021).

Muscle portions put into vibration increase the sensitivity of muscle spindles and increase muscle stiffness to dampen the vibration. Consequently, the activity of α-motoneurons in the muscle and the number of actin-myosin cross-bridges already formed increase (Barrera-Curiel et al., 2019). Therefore, the time required to absorb the loosening of the elastic component in series can be significantly reduced, decreasing the overall time between stimulus perception and the response involving force development. This could account for our results in reduced reaction times found in the VG after the 5-minute WBV warm-up but not in the CG (Table 2). There is little recent literature regarding the acute effect of WBV on reaction time. However, our results agree with previous studies. Maden et al. found that WBV improved reaction time (Maden et al., 2024); however, they refer to the upper extremity. Also, although the protocol was of the same duration as ours (5 min), the continuous stimulation time with WBV was 1 min alternating to 1 min rest. However, anatomical and functional differences exist between the upper and lower extremities in the central nervous system activation (Li et al., 1998). Bertozzi et al. applied a WBV protocol to the lower limbs and showed no effect of WBV frequency on reaction time (28); however, the administered protocol had a low frequency between 2–10 Hz administered for 210 s. In contrast, Kim et al. demonstrated an improvement in reaction ability with a protocol including a progressive frequency starting at 26 Hz (Kim, 2018). This underscores how important the training protocol is and how important it is to standardize protocols in characteristics of the exercises performed on the platform and in vibration frequency to have certain and homogeneous results. However, improvements in reaction time were observed in both the VG and SVG, with no significant between-group differences. This suggests that the dynamic warm-up exercises, which included rapid posture changes and preparatory movements, contributed to enhanced neuromuscular readiness and reaction ability, independent of vibration exposure.

The activation of the primary endings of the muscle spindle following WBV is demonstrated to stimulate the stretch reflex, promoting contraction of agonist muscles and inhibition of antagonist muscles through the activation of the Ia inhibitory interneurons (Barrera-Curiel et al., 2019). Consequently, better intramuscular coordination lowers the braking force around the hip and back, key body segments in SRT performance. This would justify the improvement in the SRT score. In our study, the significant improvement in SRT performance over time between the two measurements occurred in all three groups (Table 2). In line with our results, Cochrane et al. showed improved flexibility measures following the WBV protocol involving the arm and leg. Unlike our study, this increase was significant in the VG but not in the control group, and the group performed other exercise protocols (Cochrane et al., 2010). The SRT administered by Cochrane et al. had been run twice each detection (4 times in total), in contrast to our three times per detection (six times in total). In a study conducted earlier by our research group, performing SRT six times significantly improved the SRT score in different conditions, demonstrating a strong learning effect and improved performance in flexibility tests due to the warm-up factor and, consequently, the threshold of the firing rate of the muscle spindle after a higher number of SRT repetitions (Petrigna et al., 2025b). This could justify the SRT improvement we found in all groups.

**FIGURE 5:**
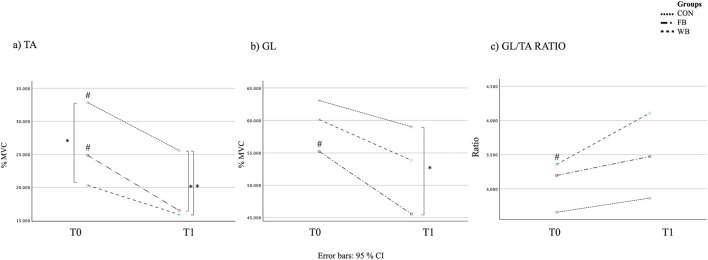
Interactions between group and time for **(a)** Tibialis Anterior (TA MVC %); **(b)** Gastrocnemius Lateralis (GL MVC %); **(c)** agonist/antagonist muscle ratio (GL/TA ratio). Data are expressed as mean ± SD. # = p < 0.05 within-group (T0 vs. T1), * = p < 0.05 between groups (CG vs. VG and CG vs. SVG). VG (vibration group), SVG (sham vibration group), CG (control group), MVC (Maximum voluntary contraction).

To our knowledge, the only published study on the effects of WBV on RSI is a recent study from 2024 administering a single bout of WBV whose results agree with ours, but the participants had a history of primary, unilateral anterior cruciate ligament reconstruction. In Dewig et al.’s study (Dewig et al., 2024), the VG and the SVG showed a non-significant decrease in RSI. In our study, just the CG had a decreased RSI between T0 and T1. RSI score derived from a drop jump (DJ) performance. DJ produces higher peak torque and more rapid torque development by the plantar flexor muscles than that by the knee and hip extensors compared to CMJ (Ando et al., 2021). In addition, compared to CMJ, the DJ has more complex performance patterns, like balance and impact components. Indeed, many factors could influence DJ performance, presenting higher variability between and within-subjects, like attentional focus strategy (Furuhashi et al., 2023), landing strategy (Kuntze et al., 2021), and Muscle-Tendon Stiffness (Gervasi et al., 2022). Consequently, it is reasonable to assume that the modification and improvement of all these components and, thus, improvement of the DJ performance might be a slower process than the others. However, WBV did not cause significant deterioration as it did in the CG (Table 2). As mentioned earlier, WBV stimulates the TVR response. However, it is known that if muscle spindles are stimulated with vibration for a long period, they will show signs of fatigue (i.e., reduced EMG activity) (Torvinen et al., 2002). Torvinen et al. showed a decreased power EMG signal frequency in the vastus lateralis and gluteus medius muscles after the vibration intervention, indicating muscle fatigue, particularly in the hip region, accompanied by a physical performance decrease (Torvinen et al., 2002). Exploratory sEMG analysis revealed changes in muscle activation patterns immediately after the vibration protocol, specifically indicated by a decrease in TA, GL activation and GL/TA ratio (significant for GL and GL/TA ratio, Table 4) (Figures 5a,b). While these changes may reflect neuromuscular adjustments, they should be interpreted as associative rather than mechanistic evidence. Several studies agree with our results, but do not confirm the association between decline in physical performance and a decrease muscle activation assessed by sEMG (McBride et al., 2010; Avelar et al., 2012). In fact, the significant decrease in EMG signal activation of the GL or the different activation GL/TA ratio did not affect performance, which improved at T1 in the VG group in our study (Table 2). Avelar et al. finds improved physical performance independent of increased electromyographic activation; In the study the sprint performance increases after WBV was not accompanied by increased electromyography activation of the vastus lateralis (Avelar et al., 2012); Cormie et al. reported an increase in vertical jumps performance after acute WBV with no change in sEMG activity (Cormie et al., 2006); McBride et al., in which the authors reported that the addition of WBV to the squat increased the maximal muscular strength of the triceps sure muscles not accompanied by a significant increase in muscle activation (McBride et al., 2010).

Beyond thermal effects, the structure of the warm-up protocol may have induced a state of post-activation potentiation (PAP), A possible mechanism of action proposed by Avelar et al. to explain the improvement in muscle performance independent of the change in sEMG signal (Avelar et al., 2012). The inclusion of high-intensity, bodyweight exercises like maximal-effort repetitions performed in a dynamic manner can serve as a conditioning stimulus. PAP occurs when the muscle does a high-intensity contractile activity before physical performance, and the following muscle performance will be improved. This is due to the phosphorylation of myosynergic light chains and the increased recruitment of motor units at a high activation threshold (Blazevich and Babault, 2019; Seitz and Haff, 2016). Although our protocol did not employ heavy external loads, the maximal voluntary effort during dynamic movements could have elicited a potentiating effect. The subsequent 1-minute recovery period before post-testing likely allowed for the expression of this PAP, contributing to the improved jump performance in the VG and SVG, a mechanism supported by contemporary warm-up strategies for explosive tasks (Silva et al., 2018).

When the vibratory stimulus is found to be adequate, this stimulus could predispose the occurrence of a PAP (Tillin and Bishop, 2009). However, the observed effects in the VG should be interpreted as the result of the combined warm-up protocol (structured exercises performed concurrently with vibration), not vibration in isolation. Since the SVG, which performed the identical exercises without vibration, achieved comparable gains in CMJ, RT, and SRT, the exercise component appears to be the primary driver of the acute performance enhancements. The role of WBV in this context remains unclear from our data, as it did not statistically augment the effects of the exercise-based warm-up. While the literature confirms that both thermal and neuromuscular mechanisms underpin warm-up efficacy (McGowan et al., 2015), our results indicate that the addition of WBV at 45 Hz did not provide a statistically significant augmentation over the dynamic exercise component alone. The identical improvements in CMJ, reaction time, and sit-and-reach performance in the SVG demonstrate that the exercise regimen itself was sufficient to elicit acute adaptations. This suggests that in a young, active population, the critical element for enhancing lower-limb power is the performance of dynamic, sport-specific movements that raise core temperature and prime the neuromuscular system, rather than the specific modality of concurrent vibratory stimulation.

This study has limitations: Although a single practice trial was provided for each test before data collection to minimize initial learning effects, the proximity of T0 and T1 within a single session means that some residual familiarization or neuromotor adaptation cannot be entirely excluded. However, this procedural step strengthens confidence that the observed changes are more likely attributable to the intervention than to initial task novelty. Full blinding of participants was not feasible due to the nature of the WBV intervention. The lack of full participant blinding may have introduced performance bias, as participants in the vibration group might have been motivated by the sensory feedback, potentially enhancing placebo-driven performance improvements. However, the use of a sham group with identical equipment but no vibration helped control for this confounding factor. Completing both physical evaluation sessions and the MVC series in a single day has the potential to cause fatigue; this would influence physical performance and EMG amplitude. However, we considered a long recovery time, and the three groups had the same condition. The literature regarding WBV is too varied in the protocols administered and parameters analyzed; this limits the comparison of our results with those of others. At the same time, the presence of a control group and a second experimental group reduces the potential bias in the results obtained. Future studies could test the effectiveness of the same protocol before and during real sports competitions.

## 5 Conclusion

In our study, exploratory sEMG data revealed neuromuscular changes that coincided with performance improvements, but these findings should be interpreted as associative rather than mechanistic. The relationship between performance improvement and muscle electrical activity due to WBV should be further investigated. In conclusion, a 5-minute dynamic warm-up protocol effectively improved or maintained performance in sport-related tests of power, reaction time, and flexibility in young, active males. The addition of whole-body vibration to this protocol did not provide statistically significant additional benefits over the same warm-up performed without vibration but No immediate adverse effects, such as performance decline, were observed during the WBV. Therefore, the acute performance enhancements can be attributed to the exercise component of the warm-up. Future research should investigate whether WBV may offer advantages in different populations, over longer durations, or in real-world sport-specific scenarios.

## Data Availability

The raw data supporting the conclusions of this article will be made available by the authors, without undue reservation.
